# Investigation on the Use of Traditional Chinese Medicine for Polycystic Ovary Syndrome in a Nationwide Prescription Database in Taiwan

**DOI:** 10.3390/jcm7070179

**Published:** 2018-07-22

**Authors:** Wan-Ting Liao, Jen-Huai Chiang, Chia-Jung Li, Ming-Tsung Lee, Cheng-Chiung Su, Hung-Rong Yen

**Affiliations:** 1 Graduate Institute of Chinese Medicine, School of Chinese Medicine, College of Chinese Medicine, China Medical University, Taichung 404 Taiwan; enolainsky@gmail.com; 2 Department of Chinese Medicine, Show Chwan Memorial Hospital, Changhua 500, Taiwan; 3 Management Office for Health Data, China Medical University Hospital, Taichung 404, Taiwan; zinvii@gmail.com; 4 College of Medicine, China Medical University, Taichung 404, Taiwan; 5 Research Assistant Center, Show Chwan Memorial Hospital, Changhua 500, Taiwan; nigel6761@gmail.com (C.-J.L.); lee6717kimo@yahoo.com.tw (M.-T.L.); 6 Post Baccalaureate Medicine, Kaohsiung Medical University, Kaohsiung 807, Taiwan; yakultaxel@gmail.com; 7 Department of Chinese Medicine, China Medical University Hospital, Taichung 404, Taiwan; 8 Research Center for Traditional Chinese Medicine, Department of Medical Research, China Medical University Hospital, Taichung 404, Taiwan; 9 Research Center for Chinese Herbal Medicine, China Medical University, Taichung 404, Taiwan; 10 Chinese Medicine Research Center, China Medical University, Taichung 404, Taiwan; 11 Department of Biotechnology, Asia University, Taichung 413, Taiwan

**Keywords:** Polycystic ovary syndrome, traditional Chinese medicine, Jia-Wei-Xiao-Yao-San

## Abstract

Polycystic ovary syndrome (PCOS) is a common condition, affecting 5–10% of women of reproductive age worldwide. It has serious reproductive implications and causes mood disorders and metabolic disorders, such as type-2 diabetes. Because PCOS reflects multiple abnormalities, there is no single drug that can treat all its symptoms. Existing pharmaceutical agents, such as oral contraceptives (OCs), are suggested as a first-line therapy for menstrual irregularities; however, OCs are not appropriate for women pursuing pregnancy. Additionally, insulin-sensitizing agents, which appear to decrease insulin levels and hyperandrogenemia in women with PCOS, have been associated with a high incidence of gastrointestinal adverse effects. It is a common practice in Chinese society to receive traditional Chinese medicine (TCM) for treatment of gynecological problems and infertility. Current research demonstrates that several herbs and herbal formulas show beneficial effects in PCOS treatment. In this study, we conducted the first large-scale survey through the Taiwan National Health Insurance Program database to analyze TCM utilization patterns among women with PCOS in Taiwan during 1997–2010. The survey results revealed that 89.22% women with newly diagnosed PCOS had received TCM therapy. Jia-Wei-Xiao-Yao-San and Xiang-Fu (Rhizoma Cyperi) were the most commonly used formula and single herb, respectively, in the database. In addition, we found that the top five commonly prescribed single herbs and herbal formulas have shown promise in treating symptoms associated with PCOS.

## 1. Introduction

Polycystic ovary syndrome (PCOS) is a common condition in humans, affecting 5–10% of women of reproductive age worldwide [1]. It has serious reproductive implications, such as anovulatory infertility, oligomenorrhea, amenorrhea, hyperandrogenism, and pregnancy complications [2]. It can also lead to mood disorders—according to a review report, the prevalence of depression among women with PCOS is 4 times greater than that among women without PCOS [3]. Research over the last few decades has revealed that PCOS is strongly associated with metabolic disorders, including an increased risk of insulin resistance, type-2 diabetes, obesity, and cardiovascular diseases (worsening of lipid profile and blood-vessel function; high blood pressure) [4]. Some reports have suggested that women with PCOS might have an increased prevalence of nonalcoholic fatty liver disease (NAFLD) and high C-reactive protein levels [5,6,7].

Because PCOS reflects multiple abnormalities, current treatment protocols for PCOS aim to achieve different goals, including healthy weight control, amelioration of hyperandrogenic symptoms, management of underlying metabolic and reproductive complications, and improvement of quality of life. In PCOS, weight management through lifestyle and behavioral intervention is a first-line treatment strategy recommended by evidence-based guidelines [8,9]. With regard to pharmaceutical treatments, oral contraceptives (OCs) are suggested as a first-line therapy for menstrual irregularities and hyperandrogenism [10,11]. However, OCs are not appropriate for women pursuing pregnancy, and they might cause weight gain, thus exacerbating the PCOS [12]. For fertility needs, clomiphene induces ovulation through the release of the gonadotropin-releasing hormone and, subsequently, gonadotropin from the anterior pituitary. However, pregnancy rates in clomiphene therapy have remained low among overweight women with PCOS [13,14,15]. Management approaches for metabolic disorders include treatment with metformin and thiazolidinediones, which appear to decrease insulin levels and hyperandrogenemia in women with PCOS [16]. However, use of metformin is associated with a high incidence of gastrointestinal adverse effects [17]. Moreover, because of the possibility of cardiovascular adverse events, thiazolidinediones are not suggested for nondiabetic women with PCOS [18,19].

It is a common practice in Chinese society to receive treatment with herbal remedies or acupuncture for obstetrical and gynecological conditions, such as uterine fibroids, dysmenorrhea, climacteric syndrome, premenstrual syndrome, and infertility. Previous studies have reported that, in women with PCOS, treatment with Chinese herbal medicine in conjunction with clomiphene citrate provides higher pregnancy rates than treatment with clomiphene citrate alone [20,21,22,23]. Moreover, acupuncture therapy might increase menstrual frequency and ovulation rates [24,25,26,27,28]. Research in diabetes and insulin resistance has shown progress in recent years. Integrating traditional Chinese medicine (TCM) healthcare into diabetes care has been shown to decrease the risk of developing kidney failure and stroke in type-2 diabetes mellitus [29,30]. Therefore, we hope to understand the clinical characteristics and treatment behavior of women with PCOS with regard to TCM.

In Taiwan, TCM has been reimbursed under the current Taiwanese National Health Insurance (NHI) system since 1996. At the end of 2010, approximately 99.89% of the total 23 million population were enrolled in the NHI [31]. Although the broadly defined category of TCM includes Chinese herbal medicine, proprietary Chinese medicine, acupuncture, moxibustion, manipulation, mediation, Qi management, and other practices, the NHI program covers only three major modalities: (1) Chinese herbal products (CHPs) manufactured by GMP (good manufacturing practice)-certified pharmaceutical companies (e.g., concentrated scientific TCM granules and finished herbal products); (2) acupuncture, moxibustion, and cupping therapy; and (3) manipulative therapy (including acupressure, chiropractic, and tui na massage) [32]. All data regarding the use of TCM in Taiwan have been collected in the National Health Insurance Research Database (NHIRD). However, there is a lack of large-scale statistical data on the clinical usage of TCM in PCOS. Therefore, this study aimed to understand the TCM prescription patterns and combinations among women with PCOS in Taiwan in order to gain information for further pharmacologic experiments and clinical trials.

## 2. Materials and Methods

### 2.1. Data Source

This study used reimbursement claims data deposited in the NHIRD, which has been implemented by the Taiwan government since 1996. The NHIRD contains longitudinal data of a cohort comprising 1 million participants randomly selected from among insurance beneficiaries between January 1997 and December 2013 (LHID2000). These datasets include information on patient characteristics; date of clinic visit; diagnosis codes in accordance with the International Classification of Diseases, Ninth Revision, Clinical Modification (ICD-9-CM) system; prescription details for Chinese herbal medicine; and detailed claims data for examination and disease management for all admitted patients and outpatients. In this study, NHRI datasets from 1997 to 2010 were selected for use as the research database, and patients were followed to December 2013. This study was approved by the institutional review board of China Medical University in central Taiwan (CMUH104-REC2-115).

### 2.2. Study Design and Population

**Figure 1:**
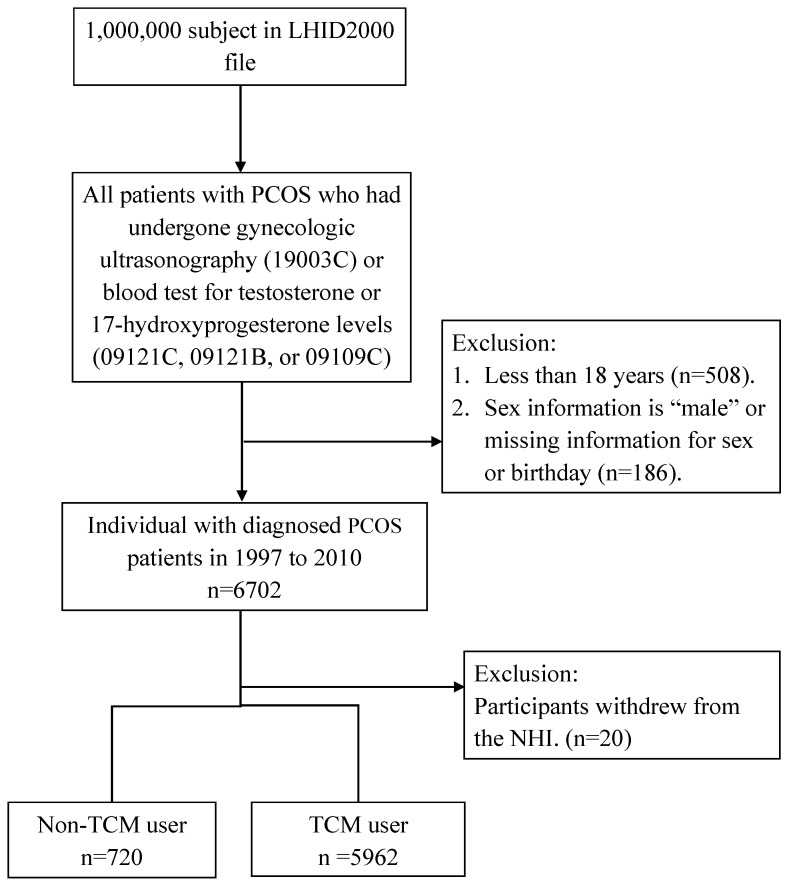
Flowchart of subjects from the one million samples randomly selected from the National Health Insurance Research Database (NHIRD). TCM: traditional Chinese medicine; PCOS: polycystic ovary syndrome; NHI: National Health Insurance.

The flowchart for selection of PCOS cases is shown in Figure 1. This study included patients who were diagnosed with PCOS (ICD-9-CM code: 256.4) in NHIRD from 1 January 1997 to 31 December 2010. All cases had undergone gynecologic ultrasonography (19003C) or blood testing for testosterone or 17-hydroxyprogesterone levels (09121C, 09121B, or 09109C) within a year of diagnosis. We excluded the beneficiaries less than 18 years old (*n* = 508), if sex information indicated male, or if there was missing information for sex or birthday (*n* = 186). At last, participants who withdrew from the NHI were also excluded from our study (*n* = 20). Patients who had been receiving TCM between the date of initial diagnosis of PCOS and the study endpoint were included in the TCM users group (*n* = 5962). The first diagnosis date of accepted TCM after initial PCOS diagnosis date was considered the index date; patients with PCOS who had not taken any TCM after the initial PCOS diagnosis date were included in the non-TCM cohort (*n* = 720).

### 2.3. Potential Confounders

We identified any potential confounders for PCOS diagnosed before the index date, including the following comorbidities: diabetes mellitus (ICD-9-CM: 250.x), infertility female (628.x), hirsutism (704.1), acne varioliformis (706.0 or 706.1), obesity (278), lipid metabolism disorders (272.0, 272.1, 272.2, 272.3 or 272.4), major depression (296.2x or 296.3x), anxiety (300.x), and amenorrhea (626.0 or 626.1).

### 2.4. Statistical Analysis

Mean values and standard deviation were described for continuous variables, and percentages were described for categorical variables. Intergroup comparison was performed by the Student t-test and chi-square test for continuous and categorical variables, respectively. Statistical analysis was performed using SAS 9.4 (SAS Institute, Cary, NC, USA) software. Two-tailed *p* values < 0.05 indicated statistical significance.

## 3. Results

### 3.1. Subject Characteristics

The present analysis included 6682 subjects who were newly diagnosed with PCOS during 1997–2010 (Table 1). All enrolled patients had undergone gynecologic ultrasonography or blood testing for serum testosterone levels. Among the enrolled subjects, while 720 did not receive TCM for treatment of PCOS in the follow-up period, 5962 had received TCM for PCOS treatment. In both cohorts (TCM users and nonusers), the highest proportion of patients were in the age group of 18–29 years. There was no substantial difference in urbanization level or standard of hospitals (where PCOS was diagnosed) between the two groups. With regard to comorbidities, TCM users had a higher prevalence of infertility (*p* = 0.027), amenorrhea (*p* < 0.0001), and anxiety (*p* = 0.0002) than TCM nonusers. The prevalence of diabetes mellitus, obesity, lipid metabolism disorders, and major depression was similar in both groups.

### 3.2. Distribution of Types of TCM

The results of analysis of medical records in the TCM outpatient departments revealed that 50.37% of patients with PCOS were treated with herbal medicine only, 0.18% with acupuncture or manipulative therapy only, and 49.45% with combination treatment. With regard to the frequency of hospital visits, most patients (60.32%) had visited TCM clinics less than three times. Approximately 25.9% of the patients had received TCM treatment more than six times (Table 2).

### 3.3. TCM Prescription Patterns for PCOS

We conducted an analysis to investigate the prescription patterns of CHPs and identified 10 of the most commonly prescribed Chinese herbal formulas and single herbs (Table 3). The most commonly prescribed Chinese herbal formula was Jia-Wei-Xiao-Yao-San (JWXYS; Supplemented Free Wanderer Powder), followed by Gui-Zhi-Fu-Ling-Wan (GZFLW; Cinnamon Twig and Poria Pill), and Dang-Gui-Shao-Yao-San (Lesser Abdomen Stasis-Expelling Decoction). The most commonly prescribed single herb was Xiang-Fu (*Cyperus rotundus* L.), followed by Da-Huang (*Rheum officinale* Baill.) and Yi-Mu-Cao (*Leonurus artemisia* (Lour.) S. Y. Hu).

### 3.4. Frequency Distribution of Disease Categories in TCM and Non-TCM Visits

We analyzed the frequency distribution of disease categories for TCM and non-TCM visits on the basis of ICD-9-CM codes from the claims data. Among TCM users, genitourinary diseases (99.21%) were the most common reasons for visiting TCM clinics, followed by digestive (99.14%) and respiratory (99.03%) disorders. In case of TCM nonusers visiting Western medical clinics, digestive disorders (95.42%) were more frequent than genitourinary diseases (94.58) and respiratory disorders (94.44%). The disease patterns among TCM users who visited clinics were similar to those among TCM nonusers (Table 4).

## 4. Discussion

In 2012, the proceedings of a National Institutes of Health (NIH) workshop suggested that the Rotterdam 2003 criteria be used for diagnosis of PCOS. The Rotterdam criteria recommended that PCOS be diagnosed on the basis of the presence of two of the following three features: oligo- and/or anovulation, clinical and/or biochemical hyperandrogenism, and polycystic ovaries identified by ultrasonography. Thus, in the present study, we selected cases from the NHIRD on the basis of examination codes indicating gynecologic ultrasonography and blood testing for testosterone level.

This study is the first large-scale report on the utilization patterns of TCM by patients newly diagnosed with PCOS. It involved analysis of claims data from TCM and non-TCM clinic visits covered by the NHI in Taiwan. According to a previous study involving the general population in Taiwan [33], 31.2% of patients used TCM in 2010, with 59.3% of TCM users being women. The study also found that herbal remedies were the most commonly used therapeutic approach in TCM (58.2%), with the frequency of combination therapy with herbal remedies being 3.8%. The present findings revealed that patients with PCOS had a relatively high tendency to consult TCM practitioners (89.22%). This might be attributable to the limited efficacy or adverse effects of current management options for PCOS in Western medicine. The use of combination therapy was also substantially high among patients with PCOS in our study (49.45%; Table 3). A recent animal study showed that combination treatment with Chinese medicinal herbs and acupuncture enhances the curative effects of the former in PCOS treatment, and that acupuncture has a positive effect on improving the absorption of herbal extracts [34].

In the present study, the most common herbal formula used for PCOS treatment was found to be JWXYS, a classical TCM formula containing 10 herbs. The formula was originally recorded in *Nei Ke Zhai Zhe Yao* (Internal Medicine Abstract), written by imperial physician Xue Ji during the rule of the Ming dynasty in ancient China. In modern clinical therapy, JWXYS plays a vital role in treatment of anxiety, irritability, stress, depression, premenstrual tension, climacteric syndrome, and infertility [35,36]. In animal models, JWXYS appears to have antidepressant effects; in a previous study, JWXYS, at 20 times its standard dose, reversed the impairment of neurogenesis in the hippocampus of stressed rats [37]. Another study found that JWXYS increases plasma tumor necrosis factor (TNF)-α levels in depressed menopausal patients [38]. In a previous randomized controlled trial, JWXYS was found to show a certain effect on improving the quality of life and alleviating depressive and obsessive-compulsive behavior, phobic anxiety, and somatic symptoms in patients with generalized anxiety disorder [39].

The second most common herbal formula for PCOS treatment among the present study population was GZFLW, which has been widely used in Asiatic countries for centuries. The formula was originally recorded in *Shang Han Za Bing Lun* (Treatise on Cold Pathogens and Miscellaneous Diseases), written by Zhang Zhong jing at the end of the Eastern Han dynasty between 200 and 205 AD. It is a formula used to invigorate blood, transform blood stasis, and reduce fixed abdominal masses [40]. In previous animal studies, GZFLW was found to exert beneficial effects on symptoms associated with type-2 diabetes. In rats with diabetes, GZFLW was demonstrated to exert protective effects against vascular injury [41,42] and delay the development of diabetic nephropathy [43]. The formula also causes a significant decrease in serum total cholesterol and triglycerides and hepatic total cholesterol levels. Additionally, GZFLW has a favorable effect on impaired glucose metabolism in type-2 diabetes by improving glucose intolerance, and it has been suggested that some of this effect is derived from the reduction of TNF-α content in skeletal muscle [42]. It has also been shown to decrease blood glucose and serum urea nitrogen and creatinine levels in rats with diabetic nephropathy. Moreover, histological findings have shown that GZFLW inhibits the development of glomerular lesions, including diffuse, nodular, fibrin-cap, capsular-drop lesions, and arteriolar hyalinosis [43]. In a small retrospective study, GZFLW treatment led to a significant improvement in liver injury outcomes and blood cholesterol levels in all patients with NAFLD [44].

The third most commonly prescribed herbal formula for PCOS treatment among the present study population was Dang-Gui-Shao-Yao-San. It has been found to suppress uterine contractions through antagonistic action on both prostaglandin F2α and acetylcholine [45] and can be used to treat dysmenorrhea and abdominal pain. Wen-Jing-Tang (WJT), the fourth most commonly prescribed herbal formula for PCOS treatment (Table 3), is traditionally used to treat amenorrhea, excessive bleeding, and infertility during menses due to deficiency-cold in the Chong and Ren channels, which leads to Qi and blood stagnation. Clinically, WJT has also been shown to cause a significant decrease in plasma luteinizing hormone (LH) levels in anovulatory patients with high plasma LH concentrations, including those with PCOS [46]. Furthermore, WJT improves the functioning of human granulosa cells in vitro by enhancing 17β-estradiol and progesterone secretion [47].

The fifth most common herbal formula prescribed for PCOS treatment was Ma-Zi-Ren-Wan (MZRW), also known as hemp seed pill. According to the findings of modern pharmaceutical studies, MZRW has shown to be effective and safe for alleviating the symptoms of functional constipation [48,49,50]. In an 18-week prospective, randomized, double-blind, placebo-controlled clinical study involving 120 patients with constipation, MZRW was found to increase complete spontaneous bowel movement and decrease straining at evacuation, without any serious adverse effects [48].

In the case of single herbs, Xiang-Fu was the most commonly prescribed herb for PCOS treatment among the present study population. It has been reported to exhibit antidepressant activity in the forced swimming test in experimental animal models [51]. Da-Huang, the second most commonly prescribed single herb, contains abundant emodin, which has been proposed as a possible treatment for type-2 diabetes and other metabolic disorders. This effect is likely associated with activation of the peroxisome proliferator-activated receptor-γ and adenosine monophosphate-activated protein kinase (AMPK) signaling pathways [52,53,54,55,56,57]. Wang et al. demonstrated that, over long-term treatment, emodin (3 μM) is a potent and selective inhibitor of 11β-hydroxysteroid dehydrogenase type 1 and can ameliorate metabolic disorders in mice with diet-induced obesity [58]. In hypercholesterolemic rats, emodin has shown hypocholesterolemic effects that might be mediated by its ability to bind bile salts and subsequently increase the expression levels of cholesterol 7α-hydroxylase [59].

Yi-Mu-Cao, also known as Chinese motherwort, was the third most commonly prescribed single herb for PCOS treatment. Traditionally, Yi-Mu-Cao has been described to play a role in promoting blood flow to regulate menstruation. The herb can be used to treat menstrual disorders. In a modern animal study on diabetic mice, treatment with motherwort extract SCM-198 (4-guanidino-*n*-butyl syringate) for 3 weeks caused a decrease in fasting blood glucose and plasma triacylglycerol levels and an increase in high-density lipoprotein and plasma insulin concentrations. The extract was reported to exhibit anti-inflammatory activity and have an ameliorating effect on diabetic symptoms through inhibition of the nuclear factor-κB/IκB kinase pathway [60]. In atherosclerotic rabbits, SCM-198 has been shown to ameliorate the progression of atherosclerotic lesions by inhibiting inflammatory factors and oxidative stress [61]. Leonurine—another extract of motherwort—has also been found to have an antidepressant effect in mice, effectively restoring 5-hydroxytryptamine, noradrenaline, and dopamine levels in the hippocampus and prefrontal cortex and ameliorating hippocampal neuronal damage in mice with chronic mild stress [62]. Yan-Hu-Suo (*Corydalis yanhusuo* W. T. Wang), the most common single herb used for treating dysmenorrhea, has been reported to demonstrate significant analgesic effects in human clinical studies [63,64]. A previous study described a compound in Yan-Hu-Suo—dehydrocorybulbine—which showed antinociceptive effects owing to its interaction with dopamine-2 receptors [65].

The fifth most common single herb used for PCOS treatment was Dan-Shen, the dried root of the plant *Salvia miltiorrhiza* Bge. The main active components in Dan-Shen injection are danshensu, salvianolic acids A and B (SalA and B), rosmarinic acid, and prolithospermic acid [66]. Recent studies have reported that SalB could potentially be useful for treatment of insulin resistance, obesity, and type-2 diabetes [67,68,69,70,71]. Additionally, SalB has demonstrated the ability to protect pancreatic beta cells [71]. Both SalB and A have also shown hepatoprotective effects in several clinical trials on NAFLD [72,73,74,75,76,77].

From these results, we have a preliminary understanding of the usage and prescription patterns of TCM for PCOS treatment. Many among these prescription herbs have shown hepatoprotective effects, as well as beneficial effects in treatment of hyperglycemia, insulin intolerance, dyslipidemia, and anovulation. We list the possible pharmacological effects or mechanism of the herbal formulas and single herbs in Table 5. These findings indicate that Chinese herbal medicine might serve as a potentially effective therapeutic option for PCOS. Therefore, future studies should analyze the association between TCM intervention and PCOS symptoms, such as menstrual regularity, hirsutism, acne, obesity, depression, low ovulation, pregnancy, and live-birth rates. In addition to symptom assessment, serological markers can also be listed among analytical indicators for assessing the benefits of Chinese medicine in PCOS treatment.

The present study has the following strengths. First, confirming the prescription patterns of TCM through the health insurance database helped avoid the possibility of memory bias associated with questionnaire-based investigation. Second, all subjects enrolled in this study had undergone blood testing or gynecologic ultrasonography before being diagnosed with PCOS by a qualified physician. However, there are several limitations to this study. Details regarding disease severity and treatment efficacy were not known because of the lack of imaging and laboratory data in the NHIRD. Moreover, Chinese herbal decoctions or remedies that were purchased directly from TCM herbal pharmacies were not covered by NHI, which might have led to underestimation of the frequency of TCM use. However, because the NHI program allows Chinese herbal remedies to be availed at a relatively low cost, the proportion of patients taking herbal medicine outside the NHI system is relatively low. Moreover, patients might be prescribed other Western medicines or self-purchase supplements for treating PCOS or other diseases during the follow-up period. The possibility of interaction of different types of medicines also requires longer following and analysis.

## 5. Conclusions

This study is the first large-scale survey to analyze TCM utilization patterns among women with PCOS in Taiwan. Jia-Wei-Xiao-Yao-San was the most commonly used TCM formula, while Xiang-Fu was the most commonly used single herb in our dataset. In addition, we found that the top five most commonly prescribed single herbs and herbal formulas have shown promise in treating symptoms associated with PCOS. Further pharmacological investigations and clinical trials could be developed on the basis of the present findings.

## Figures and Tables

**Table 1:** Demographic characteristics of the patients newly diagnosed with PCOS in Taiwan in 1997–2010.

Variable	Without TCM		With TCM		*p* Value
	*n* = 720 (10.78%)		*n* = 5962 (89.22%)		
	*n*	%	*n*	%	
**Age at baseline**					0.0654 *
18–29	395	54.86	3539	59.36	
30–39	265	36.81	1991	33.39	
Older than 40	60	8.33	432	7.25	
Mean(SD)	29.77(6.93)		29.09(6.67)		0.0094 ^‡^
**Urbanization**					0.7906 *
1 (highest)	236	32.78	1972	33.08	
2	230	31.94	1824	30.59	
3	116	16.11	984	16.5	
4	89	12.36	707	11.86	
4+ (lowest)	49	6.81	475	7.97	
**Hospital level (where diagnosed with PCOS)**					0.2209 *
Medical center	170	23.64	1326	22.24	
Regional hospital	281	39.08	2172	36.43	
District hospital	118	16.41	1070	17.95	
Physician clinics	150	20.86	1394	23.38	
**Baseline comorbidity**					
Diabetes mellitus	43	5.97	441	7.4	0.1636 *
Infertility female	129	17.92	1362	22.84	0.0027 *
Hirsutism	1	0.14	27	0.45	0.3567 *
Acne varioliformis	227	31.53	1950	32.71	0.5236 *
Obesity	14	1.94	159	2.67	0.2489 *
Disorders of lipoid metabolism	42	5.83	294	4.93	0.2954 *
Major depression	16	2.22	137	2.3	0.898 *
Anxiety	60	8.33	786	13.18	0.0002 *
Amenorrhea	218	30.28	2630	44.11	<0.0001 *
Interval between onset of PCOS disease and the first TCM consultation, days mean (median)			530.88(246)		

^‡^*t* test; * chi-square test.

**Table 2:** Distribution of Chinese medicine according to type of Chinese medicine treatment received in women with PCOS, stratified by number of outpatient visits.

Number of TCM Visits (Times/per Year)	Only Chinese Herbal Remedies	Only Acupuncture or Traumatology	Combination of Both Treatments	Total of TCM (*n* = 5962)
	*n* = 3003 (50.37%)	*n* = 11 (0.18%)	*n* = 2948 (49.45%)	
	*n* (%)	*n* (%)	*n* (%)	*n* (%)
1–3	2016 (67.13)	11 (100)	1569 (53.22)	3596 (60.32)
4–6	361 (12.02)	0	461 (15.64)	822 (13.79)
>6	626 (20.85)	0	918 (31.14)	1544 (25.90)

**Table 3:** Ten most common herbal formulas prescribed.

Herbal Formula	Frequency	Number of Person-Days	Average Daily Dose	Average Duration for Prescription
			(g)	(Days)
Single Chinese herb				
Xiang-Fu (Cyperus rotundus L.)	12,375	86,491	1.0	7.0
Da-Huang (Rheum officinale Baill.)	11,350	83,520	0.7	7.4
Yi-Mu-Cao (Leonurus artemisia (Lour.) S. Y. Hu)	10,824	77,687	1.1	7.2
Yan-hu-suo (Corydalis yanhusuo W. T. Wang)	9889	68,526	1.1	6.9
Dan-shen (Salvia miltiorrhiza Bge.)	8072	61,438	1.1	7.6
Gan-Cao (Glycyrrhiza uralensis Fisch.)	7818	53,026	0.9	6.8
Tu-Si-Zi (Cuscuta chinensis Lam.)	7178	52,268	1.3	7.3
Huang-Qin (Scutellaria baicalensis Georgi)	7616	52,213	1.2	6.9
Bei-Mu (Fritillaria thunbergii Miq.)	5860	39,858	1.1	6.8
Du-Zhong (Eucommia ulmoides Oliv.)	5414	39,649	1.1	7.3
Combined Chinese herb				
Jia-Wei-Xiao-Yao-San	21,305	15,3952	4.3	7.2
Gui-Zhi-Fu-Ling-Wan	8779	62,294	3.9	7.1
Dang-Gui-Shao-Yao-San	8470	58,510	4.4	6.9
Wen-Jing-Tang	7970	58,018	4.2	7.3
Ma-Zi-Ren-Wan	6850	51,079	2.7	7.5
Chuan-Xiong-Cha-Tiao-San	5643	36,836	3.9	6.5
Gui-Pi-Tang	4895	36,050	4.3	7.4
Ger-Gen-Tang	5471	35,096	4.3	6.4
Shao-Fu-ZhuYu-Tang	4994	34,233	3.8	6.9
Xue-Fu-Zhu-Yu-Tang	4473	33,638	3.8	7.5
List according to person-days				

**Table 4:** The distribution of TCM and non-TCM users by major disease categories/diagnosis in patients with PCOS.

Disease (ICD-9-CM)	Without TCM (*n* = 720)		With TCM (*n* = 5962)		*p* Value
	*n*	%	*n*	%	
Infectious and parasitic disease (001–139)	439	60.97	4433	74.35	<0.0001
Neoplasms (140–239)	324	45.00	3384	56.76	<0.0001
Malignant (140–208)	19	2.64	257	4.31	0.0332
Benign (210–229)	313	43.47	3242	54.38	<0.0001
Endocrine, nutritional, and metabolic disease and immunity disorder (240–279)	720	100.00	5962	100.00	-
Blood and blood-forming organs (280–289)	136	18.89	1482	24.86	0.0004
Mental disorder (290–319)	156	21.67	2243	37.62	<0.0001
Nervous system (320–389)	532	73.89	5232	87.76	<0.0001
Circulatory system (390–459)	200	27.78	2415	40.51	<0.0001
Respiratory system (460–519)	680	94.44	5904	99.03	<0.0001
Digestive system (520–579)	687	95.42	5911	99.14	<0.0001
Genitourinary system (580–629)	681	94.58	5915	99.21	<0.0001
Complications of pregnancy, childbirth, and the puerperium (630–676)	309	42.92	3015	50.57	0.0001
Skin and subcutaneous tissue (680–709)	595	82.64	5553	93.14	<0.0001
Musculoskeletal system and connective tissue (710–739)	394	54.72	4964	83.26	<0.0001
Congenital anomalies (740–759)	30	4.17	455	7.63	0.0007
Certain conditions originating in the perinatal period (760–779)	122	16.94	1053	17.66	0.6329
Symptoms, signs, and ill-defined conditions (780–799)	607	84.31	5772	96.81	<0.0001
Injury and poisoning (800–999)	422	58.61	5129	86.03	<0.0001

**Table 5:** Possible pharmacological effects or mechanisms of the most common herbal formulas and single herbs for the treatment of patients with PCOS.

Chinese Herbal Products	Model/Design	Possible Pharmacological Effects or Mechanism	
Herbal formulas			
Jia-Wei-Xiao-Yao-San	Multicenter, Double blind, Placebo controlled RCT	Antidepressant effects	Improve quality of life, reduce depressive, obsessive-compulsive, somatic symptoms of generalized anxiety disorder
	Stressed rats	Antidepressant effects	Reverse the impairment of neurogenesis in the hippocampus
Gui-Zhi-Fu-Ling-Wan	Retrospective study	Anti-inflammation Hypolipidemic effects	Reduction in liver injury tests and blood cholesterol
	Diabetic rats	Anti-diabetic effects Hypolipidemic effects	Improve glucose intolerance, decrease in serum total cholesterol and TG levels Delay the development of diabetic nephropathy
Dang-Gui-Shao-Yao-San	Rats	Analgesic effect	Suppress uterine contraction through antagonistic action on both prostaglandin F2α and acetylcholine
Wen-Jing-Tang	Clinical Trial		Decrease in plasma LH level in anovulatory patients including those with PCOS
	Human granulosa cells		Enhancing 17β-estradiol and progesterone secretion
Ma-Zi-Ren-Wan	Double blind, Placebo controlled RCT		Increase complete spontaneous bowel movement and decrease straining at evacuation
Single herbs			
Xiang-Fu	Rats	Antidepressant	Had antidepressant activity in the forced swimming test
Da-Huang	Mice	Hypocholesterolemic effects	Activation of the PPAR-γ and AMPK signaling pathways
Yi-Mu-Cao	Mice	Potential to treat insulin resistance, and type-2 diabetes	Through inhibition of the NF-κB/IκB kinase pathway
Yan-hu-suo	Mice	Analgesic effects	Antinociceptive effect due to interaction with D2 receptors
Dan-shen	Rats	Potential to treat insulin resistance, obesity, and type-2 diabetes	SalB: Increased phosphorylated AMPK protein expression, GLUT 4 and glycogen synthase protein expressions
	Rats	Hepatoprotective effects	SalA: Suppressing ROS, MDA; preventing the decreased expression of SOD SalB: Through SIRT1-mediated HMGB1 deacetylation
